# Supplementary material for: Supportive consensus
Source: PLoS One. 2020 Dec 17;15(12):e0243215. doi: 10.1371/journal.pone.0243215 (PMC7746199; doi:10.1371/journal.pone.0243215)
Supplement: Attachment — Submitted filename: Response to Reviewers.pdf [file pone.0243215.s001.pdf]

November 6, 2020

Fei Chen
Academic Editor
PLOS ONE
Subject: Submission of revised paper PONE-D-20-21157, 'Supportive Consensus'.

Dear Dr. Chen,

Thank you for your e-mail dated 24th July 2020, enclosing the reviewers' comments. We have carefully reviewed the comments and have revised the manuscript accordingly. Our responses are given in a point-by-point manner below. Changes to the document are shown in blue.

We have rechecked the language, and the mistakes detected in the reading proof corrected. The paper's contribution has been improved in a new section that explains the main results and the differences between the approaches we propose. Regarding the convergence analysis, we have included a new section that demonstrates the solution in a scenario with perfect information regarding the solution obtained by the algorithms. The convergence of those algorithms is ensured by the original work of Olfati–Saber and Murray, in which we base our proposal. As the algorithms match the requirements, stability is ensured. We have highlighted this fact in the paper too. The rest of the observations have been appropriately addressed, in our opinion.

We hope the revised version is now suitable for publication and look forward to hearing from you in due course.

Sincerely,

Dr. Alberto Palomares .
Associate Professor
Universitat Politecnica de Valencia (Spain)

*Response to reviewer 1

Thank you for your review of our paper. We have answered each of your points below

1. *There are many grammatical mistakes in this paper. For example, in page 1 line 2, it should be "to deal with" instead of "to dealing with". In page 1 line 14, it should be "must obtain" instead of "must to obtain". Please check the whole paper carefully to correct all the grammatical mistakes.*
   We have rechecked the language. The mistakes indicated by the reviewer have been corrected, and some others detected in the reading proof.
2. *In page 3 line 80, the authors state the contributions of this paper in this paragraph. However, the introduction of the contributions is a little pale. I suggest presenting a more detailed introduction to the contributions here. For example, what algorithms do you propose? What are the differences between these proposed algorithms? How do you analyze the algorithms? How do you verify the effectiveness of the proposed algorithms?*
   We have included in the Scope section a broader explanation detailing the information required by the reviewer. In this part, we have described the algorithm, explain their differences in the approach to the supportive consensus problem, and the experiments to evaluate their performance and the quality of the solutions they reach.
3. *The assumptions of the proposed algorithms should be stated clearly. For example, can the proposed algorithm work under directed graphs? Is the underlying graph required to be connected? Can the proposed algorithms work under arbitrary initial conditions?*
   We have clarified these characteristics of the network in its definition. We assume that the consensus process runs over a non-directed, strongly connected network. Algorithms could be extended to the directed case but keeping the connection condition.
   "Let $G = \{V, E\}$ an undirected, strong connected graph with n nodes, $V = \{1, ..., n\}$, and e edges $E \subseteq V \times V$, where $(i, j) \in E$ if there exists a link between nodes i and j."
   Regarding the initial condition, the only consideration is that all the nodes must begin inside their ranges (see explanation after Eq. 21) We have remarked this fact.
   "In general, assuming that the initial values of all agents (t=0) are within their bounds, the network globally always (t) has the capacity to compensate the total amount of values[...]"
4. *The goals of these proposed algorithms should be stated clearly. Why do you need multiple algorithms to solve the same problem? What are the pros and cons of each algorithm?*
   We have added a paragraph at the beginning of the section 'Supportive Consensus Algorithms Proposal' explaining the goals and the reasons for the different algorithms.

'The algorithm that we have called SEA[...]'
Furthermore, we have included a section 'Algorithm Summary' summarizing the pros and cons of the proposed algorithms.

5. *Convergence properties of the proposed algorithms should be stated clearly. For example, what is the convergence rate? What are the exact convergent values?*
   We have included a new section that calculates the solution in a scenario with perfect information regarding the solution obtained by the algorithms. We have considered the centralized solution as the exact solution. The results obtained by the algorithms are approximations of this value.
   The convergence of the proposed algorithms is ensured by the original work of Olfati–Saber and Murray in which we base our proposal. As the algorithms match the requirements, stability is ensured. We have highlighted this fact in the paper, too, related to the sum conservation. 'As Algorithm conserves the sum of the initial values and fulfill the conditions of the Olfati–Saber consensus algorithm to converge,(Footnote: The convergence ...) we need to ensure that the Supportive Consensus function also conserves the sum. We check this condition in each one of the proposed algorithms.'

*Response to reviewer 2

Thank you for your comments. Our answers to your points are as follow

1. *It is suggested that the body of each graph appear with its corresponding title such Figure 4, 5, and the others.*
   Following the submission rules of PLOS, figures have to be moved to the end of the paper and keep the captions in place.
2. *It is suggested that we call them table rather than figure for the Figures 1,2,3 in the current manuscript.*
   These figures contain both the table and the figure, and as in the previous consideration. They appear at the end of the document.
3. *The definition of degree, which is used in line 117, is suggested to be provided in this work. Moreover, it is suggested that both the in-degree and the out-degree are provided.*
   We have included the definition of the degree of the network *" In graph theory, the degree of a node d_i is the number of edges that are incident to the node, and therefore $|N_i| = d_i$."*. As we assume a non-directed network, the definition of in- and out-degree are not necessary.
4. *The section number is left in line 179, ", as defined in Section ."*.
   We have included the name of the section in the reference (Background)
5. The usage of $x_i(t)$ is not appropriate in lines 182, 184 and 186. As we know, $x_i(t)$ is the state of node i, and it is not the consensus value.

We do not make any difference between the state of the node and its value and we use both as synonyms. The value $x_i(t)$ is the only information stored in the nodes. Its value is defined and bounded in equation 5.

6. *There are many redundant "=" in the equation below line 285.*
   We have removed the equal signs at the end of each line of equation 5

7. *The English should be improved since there are many grammatical problems such as "This paper presents a novel approach to dealing with..." (line 2), ", and we proof that if ..." (line 22). There is no need to indent the beginning of some paragraphs such as the paragraphs on line 119 and line 124.*
   We have rechecked the language. The mistakes indicated by the reviewer have been corrected, and some others detected in the reading proof.
